# Tensile property improvement of TWIP-cored three-layer steel sheets fabricated by hot-roll-bonding with low-carbon steel or interstitial-free steel

**DOI:** 10.1038/srep40231

**Published:** 2017-01-09

**Authors:** Jaeyeong Park, Jung-Su Kim, Minju Kang, Seok Su Sohn, Won Tae Cho, Hyoung Seop Kim, Sunghak Lee

**Affiliations:** 1 Center for Advanced Aerospace Materials Pohang University of Science and Technology, Pohang, 790-784, Korea; 2 Steel Research Group Technical Research Laboratories, POSCO, Kwangyang, 545-090, Korea

## Abstract

TWIP-cored three-layer steel sheets were newly fabricated by hot rolling of TWIP steel sheet surrounded by low-carbon (LC) or interstitial-free (IF) steel sheets. TWIP/LC or TWIP/IF interfaces were well bonded without pores or voids, while a few pearlites were thinly formed along the interfaces. The strengths and elongation of the TWIP-cored sheets increased as the volume fraction of TWIP-cored region increased, and were also well matched with the ones calculated by a rule of mixtures based on volume fraction or force fraction. According to digital image correlation and electron back-scatter diffraction analyses, very high strain hardening effect in the initial deformation stage and active twin formation in the interfacial region beneficially affected the overall homogeneous deformation in the TWIP-cored sheets without any yield point phenomenon occurring in the LC sheet and serrations occurring in the TWIP sheet, respectively. These TWIP-cored sheets can cover a wide range of yield strength, tensile strength, and ductility levels, *e*.*g*., 320~498 MPa, 545~878 MPa, and 48~54%, respectively, by controlling the volume fraction of TWIP-cored region, and thus present new applications to multi-functional automotive steel sheets requiring excellent properties.

Highly deformable steels such as TRansformation Induced Plasticity (TRIP) and TWinning Induced Plasticity (TWIP) steels have been actively developed in worldwide automotive industries to decrease CO_2_ emissions and increase fuel efficiency^1^,^2^,^3^,^4^. In TWIP steels, mechanical twins gradually reduce the effective mean free path of dislocations, known as dynamic Hall-Petch effect^5^, as the twin formation evolves the creation of new crystal orientations. This effect increases the flow stress, and suppresses the necking during tensile deformation by highly sustained strain hardening rate^6^,^7^. Thus, TWIP steels show high strength and ductility simultaneously^3^,^5^,^6^,^7^. In spite of excellent tensile properties, their wide commercialization in automotive applications has been postponed because they are often subjected to delayed fracture after forming^8^, liquid metal embrittlement during spot welding^9^,^10^, deteriorated surface quality due to dynamic strain aging^11^, and difficulties in carry due to paramagnetic nature of constituent phase of austenite^12^.

In order to solve aforementioned shortcomings of TWIP steels, the control of C and Mn contents, addition of Al or Si, and optimization of fabrication processes have been applied^13^,^14^,^15^,^16^. As another way, a multi-layered hybrid sheet fabrication, which is called a TWIP-cored sheet fabrication, is newly suggested in the present study. Recently developed hybrid materials such as fiber-reinforced composites, foams, and sandwich structures show newly materialized characteristics by appropriately combining unique characteristics of each material, and their fabrication techniques have been actively utilized in non-ferrous alloys^17^,^18^,^19^,^20^. In view of conventional structural steel designs, various multi-layered hybrid sheets have been fabricated to overcome a performance deadlock while keeping excellent combination of mechanical and fracture properties, *e*.*g*., to improve the ductility or toughness of martensite-based steels^21^,^22^,^23^. Nambu *et al*.^22^ reported that the tensile strength and elongation of about 950 MPa and 21%, respectively, could be obtained by the roll bonding of martensitic and stainless steel sheets. According to Inoue *et al*.^23^, the roll bonding of martensitic and low-carbon steel sheets showed the tensile strength and elongation of about 1300 MPa and 4%, respectively. Overall tensile properties are generally enhanced by achieving the excellent interfacial bonding strength, but the elongation is often seriously deteriorated by the rapid failure or delamination due to stress concentration at insufficiently bonded interfacial areas^22^. Tunnel cracks are also initiated by the rapid propagation of brittle cracks or microcracks in the martensitic steel region^23^. In the multi-layered sheet designs to obtain excellent mechanical properties based on a rule of mixtures, thus, the minimization or prevention of interfacial defects originally existed in as-fabricated sheets or subsequently formed during deformation processes is essentially needed.

Since most of shortcomings of TWIP steels are closely related with surface properties, if the surface of TWIP steel substrate is replaced by low-cost commercial steels such as low-carbon (LC) steels and interstitial-free (IF) steels, these TWIP-cored multi-layered steel sheets have merits of excellent delayed fracture properties, spot-weldability, and surface quality enough to complement drawbacks of TWIP steels. Furthermore, if the fabricated TWIP-cored sheets have a very wide range of mechanical properties by controlling the volume (or thickness) fraction of the TWIP-cored region, they can readily discover new applications for automotive structural steel sheets. Here in the present study, six TWIP-cored three-layer steel sheets containing thin LC or IF steel surface layers were fabricated by direct solid-state hot-roll-bonding using commercial hot-rolling stands. Tensile properties of these sheets as well as mono-layer TWIP, LC, and IF steel sheets were evaluated in order to obtain the basic data on applicability to vehicle body structures. Mechanisms of improved tensile properties were investigated in relation with effects of TWIP/LC or TWIP/IF interfaces on overall tensile properties by digital image correlation (DIC) and electron back-scatter diffraction (EBSD) analyses.

## Results and Discussion

### Microstructures and tensile properties

**Figure 1:**
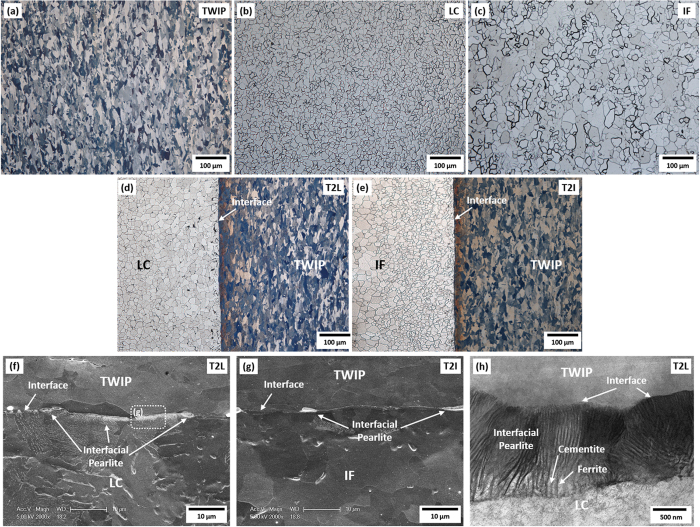
Microstructure of TWIP, LC, and IF steel sheets and T2L and T2I sheets. (**a**) Through (**e**) show optical micrographs of the TWIP, LC, and IF steel sheets and T2L and T2L sheets. (**f**,**g**) Show SEM micrographs of the TWIP/LC and TWIP/IF interfacial regions of T2L and T2I sheets, respectively. (**h**) Shows a TEM micrograph of the TWIP/LC interfacial region of T2L sheets. TWIP/LC and TWIP/IF interfaces are well bonded without pores or voids, which indicates a successful fabrication of the TWIP-cored three-layer sheets by the hot-roll-bonding. Thin elongated pearlites of about 1 μm in thickness are found along the TWIP/LC and TWIP/IF interfaces as indicated by arrows in (**f**,**g**). In the magnified TEM micrograph of the thin interfacial pearlite, most of cementite lamellae are very closely spaced, and are aligned in the direction perpendicular to the interface.

Optical micrographs of the TWIP, LC, and IF steel sheets, and the TWIP-cored T2L and T2I sheets are shown in Fig. 1(a–e). TWIP/LC and TWIP/IF interfaces are indicated by arrows in Fig. 1(d,e). These interfaces are well bonded without pores or voids, which indicates a successful fabrication of the LC/TWIP/LC and IF/TWIP/IF sheets by the hot-roll-bonding. Sizes of austenite and ferrite grains in the TWIP-cored region and LC or IF steel region, respectively, were measured by an image analyzer, and the results are shown in Table 1. The grain size is 11.7 μm in the TWIP steel sheet, and increases in the order of the LC and IF steel sheets (Fig. 1(a–c)). Ferrite grains near the TWIP/LC interface are similar to those of the LC steel (Fig. 1(d)), whereas those near the TWIP/IF interface are smaller than those of the IF steel (Fig. 1(e)). This is because the interfacial areas have the higher carbon content according to the carbon diffusion from the TWIP steel to the LC or IF steel during the hot-roll-bonding. A smaller number of carbon atoms are diffused in the TWIP/IF interface than in the TWIP/LC interface as the austenite is maintained for a shorter time during the hot-roll-bonding. Thus, carbides are hardly formed in the TWIP/IF interfacial area. Ferrite grains are also refined because the recrystallization is delayed in the carbon-diffused interfacial area by the lowered Ar_3_ temperature.

Figure 1(f–h) shows SEM and TEM micrographs of the TWIP/LC and TWIP/IF interfacial areas of the T2L and T2I sheets. A considerable number of carbides are developed in a form of film or pearlite in the LC steel region near the TWIP/LC interface of the T2L sheet according to the sufficient carbon diffusion from the TWIP steel to the LC steel during the hot-roll-bonding (Fig. 1(f)). Thin elongated pearlites of about 1 μm in thickness are also found along the TWIP/LC interface as indicated by arrows. Near the TWIP/IF interface of the T2I sheet, pearlites are also present, but their number and amount are much smaller than those of the T2L sheet (Fig. 1(g)). When these interfacial pearlites are magnified in a TEM micrograph (Fig. 1(h)), most of cementite lamellae are aligned in the direction perpendicular to the interface as cementite lamellae are grown from the interface to the LC steel interior. Cementite lamellae inside interfacial pearlites are very closely spaced, which is somewhat different from typical shapes of pearlite^24^.

**Figure 2:**
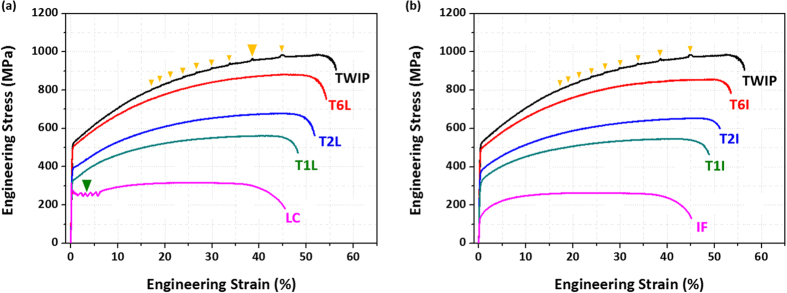
Room-temperature engineering stress-strain curves of the TWIP-cored (**a**) TL and (**b**) TI sheets as well as the TWIP, LC, and IF steel sheets. All TWIP-cored sheets are deformed without any serrations or yield point, and both the strength and elongation are approximately increased as the volume (or thickness) fraction of TWIP-cored region increases.

Figure 2(a,b) shows room-temperature engineering stress-strain curves of the TWIP, LC, and IF steel sheets, and the TWIP-cored TL and TI sheets, and the yield strength, ultimate tensile strength, and elongation are summarized in Table 1. The stress-strain curve of the TWIP sheet shows a relatively high strain hardening and serrations as marked by yellow arrows in Fig. 2(a) and its yield strength, tensile strength, and elongation are 523 MPa, 985 MPa, and 56%, respectively. The LC sheet shows the lower yield strength, tensile strength, and elongation than the TWIP sheet, together with a yield point phenomenon as marked by a green arrow in Fig. 2(a). The IF sheet has the lower strengths than the LC sheet, although it does not show the yield point phenomenon (Fig. 2(b)). All TWIP-cored sheets are deformed without any serrations or yield point (Fig. 2(a,b)). Both the strength and elongation are approximately increased as the volume (or thickness) fraction of TWIP-cored region increases. The TL and TI sheets have almost same stress-strain behavior.

**Figure 3:**
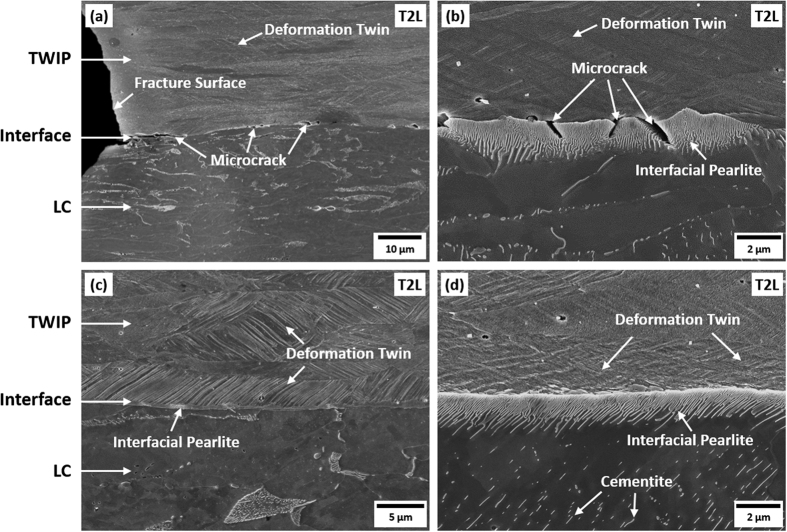
SEM micrographs of the cross-sectional area (**a**,**b**) beneath the fracture surface and (**c**,**d**) distant from the fracture surface of the T2L sheet. A few microcracks formed in thin interfacial pearlites are observable beneath the fracture surface, while they are not found in the areas distant from the fracture surface.

Figure 3(a,b) shows SEM micrographs of the cross-sectional area beneath the fracture surface of the T2L sheet. A few microcracks formed in thin interfacial pearlites are observable beneath the fracture surface because the stress is concentrated into interfacial pearlites. Figure 3(c,d) shows SEM micrographs of the cross-sectional area distant from the fracture surface of the T2L sheet. Microcracks are not found in the areas distant from the fracture surface. Since microcracks not develop into a large crack to initiate critical interfacial debonding or delamination, it is not likely that the formation of microcracks in interfacial pearlites deteriorates tensile properties. Many deformation twins are formed in the TWIP-cored region near the TWIP/LC interface.

### Tensile flow behavior – Yield point phenomenon and serrations

**Figure 4:**
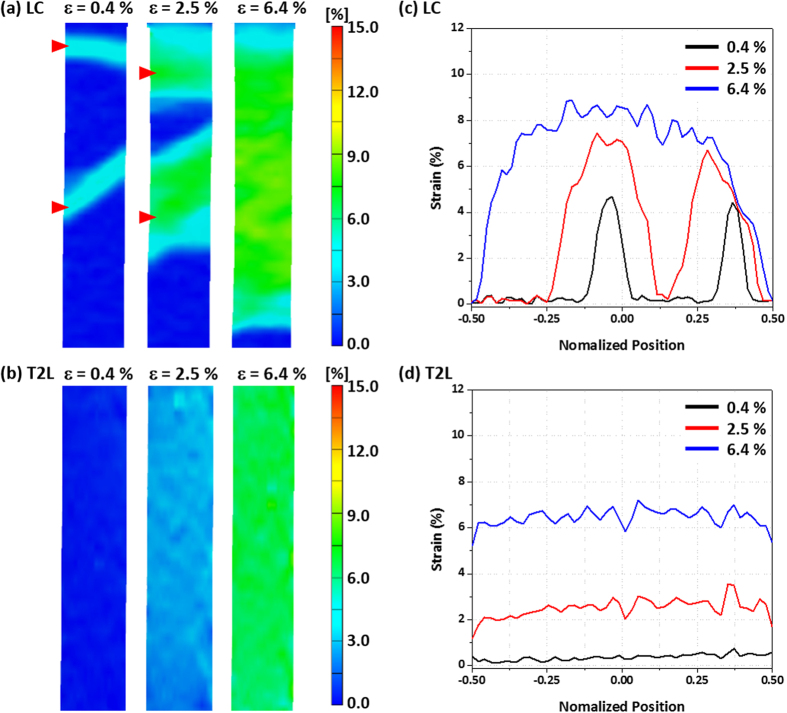
(**a**,**b**) Digital images of strain and (**c**,**d**) strain distribution curves of the LC and T2L sheets at tensile strains of 0.4%, 2.5%, and 6.4%. In the LC sheet, localized bands appear in the upper and center regions of the gage section at the strain of 0.4%, are widened at the strain of 2.5%, and are joined together to reach the almost same strain throughout the gage section at the strain of 6.4%. The T2L sheet is deformed homogeneously without any localized bands.

It is interesting to note that serrations occurring in the TWIP sheet and yield point phenomenon occurring in the LC sheet do not appear in the TWIP-cored sheets, as shown in Fig. 2(a,b). In order to investigate the serrations and yield point phenomenon, the vision strain gauge system was simultaneously used with tensile tests. Figure 4(a,b) shows digital images of strain of the LC and T2L sheets at tensile strains of 0.4%, 2.5%, and 6.4%, which correspond the yield-point strain range of the LC sheet (marked by a green arrow in Fig. 2(a)). The local strains calculated from digital images along the center line as a function of normalized position of the gage section (gage length; 25 mm) are also plotted in Fig. 4(c,d). In the normalized position, −0.5 and +0.5 indicate the two ends of the gage section, while zero means the center. The LC sheet is inhomogeneously deformed in the initial deformation stage at the strain of 0.4%, and localized bands appear in the upper and center regions of the gage section, as marked by red arrows in Fig. 4(a). At the strain of 2.5%, the localized bands are widened, and their local strains are increased. When the strain increases further to 6.4%, the two bands are joined together to reach the almost same strain throughout the gage section, which indicates the end of the yield point phenomenon and the start of homogeneous deformation. On the other hand, the T2L sheet is homogeneously deformed even in the initial deformation stage without any localized bands (Fig. 4(b–d)).

It can be expected in the TWIP-cored T2L sheet that Lüders bands might be formed in the LC region, but that the yield point phenomenon might be prevented by some peculiar deformation mechanisms. In the T2L sheet, however, localized bands are not formed at all because of very high strain hardening effect of the TWIP-cored region in the initial deformation stage as well as strong TWIP/LC interfacial bonding. Thus, the T2L sheet is continuously strain-hardened after the yielding, as shown in Fig. 2(a).

**Figure 5:**
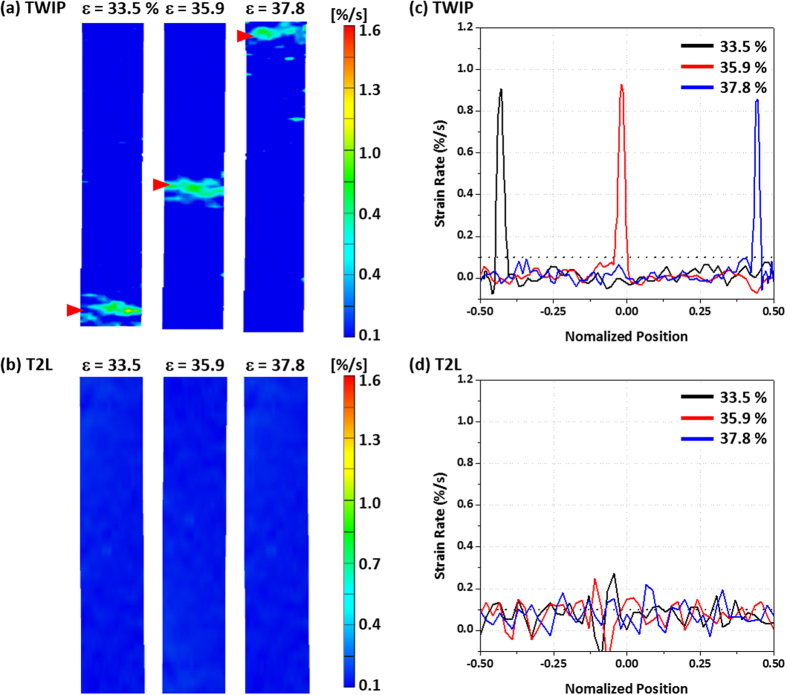
(**a**,**b**) Digital images of strain rate and (**c**,**d**) strain rate distribution curves of the TWIP and T2L sheets at tensile strains of 33.5%, 35.9%, and 37.8%. In the TWIP sheet, the localization of strain rate occurs in a form of band at the lower region of the gage section at the strain of 33.5%. This localized band moves upward as the strain increases to 35.9% and 37.8%. In the T2L sheet, the localized band is not found throughout the gage section, and the strain rate is almost same to the overall strain rate (0.1%/s) of the tensile test.

Figure 5(a,b) shows digital images of strain rate of the TWIP and T2L sheets at tensile strains of 33.5%, 35.9%, and 37.8%, which correspond the serration-appearing strain rage of the TWIP sheet (marked by a yellow arrows in Fig. 2(a)). The local strain rates calculated from digital images along the center line as a function of normalized position of the gage section are also plotted in Fig. 5(c,d). At the strain of 33.5%, the localization of strain rate occurs in a form of band at the lower region of the gage section in the TWIP sheet, as marked by a red arrow in Fig. 5(a). This localized band moves upward as the strain increases to 35.9% and 37.8%. The time of appearance of this band is well matched with the serration point (a large yellow arrow mark in Fig. 2(a)). The appearance and movement of this band can also be verified from Fig. 5(c). In the T2L sheet, the localized band is not found throughout the gage section, and the strain rate is almost same to the overall strain rate (0.1%/s) of the tensile test (Fig. 5(b–d)). These DIC analyses explain the difference in serrated and smooth stress flows in the TWIP and TWIP-cored sheets, respectively.

According to the DIC analysis data, serrations in stress-strain curve of the TWIP sheet is accompanied with the localized band formation. In order to form localized bands in the TWIP region of the T2L sheet, the higher strain is required at this band region than the other region inside the gage section. However, the TWIP/LC interface is strongly bonded in the T2L sheet, and the LC region does not have any mechanisms for accommodating the localized bands, which results in the homogeneous deformation of the T2L sheet, instead of inhomogeneous deformation, without any serrations.

**Figure 6:**
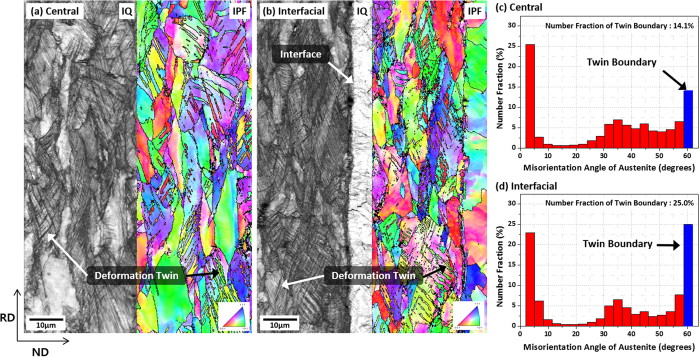
EBSD image quality (IQ) and inverse pole figure (IPF) maps and misorientation angle distributions of austenite grains in the (**a**,**c**) central TWIP region and (**b**,**d**) TWIP/LC interfacial region after the tensile fracture of the T2L sheet. Most of austenite grains in the TWIP/LC interfacial region contain many twins, and the number fraction of twin boundary is higher in the TWIP/LC interfacial region (25%) than in the central TWIP region (14.1%).

In order to more clearly explain the difference in serrated and smooth stress flows in the TWIP and T2L sheets, respectively, austenite grains of the cross-sectional areas in the central TWIP region (the region distant from the interface) and TWIP/LC interfacial region after the tensile fracture of the T2L sheet were analyzed by the EBSD. Figure 6(a–d) shows EBSD image quality (IQ) and inverse pole figure (IPF) maps and misorientation angle distributions of austenite grains. A number of twins are formed in both central TWIP and TWIP/LC interfacial regions as indicated by arrows in Fig. 6(a,b). More twins are clearly visible in the TWIP/LC interfacial region as shown in IQ maps. This result can be confirmed from the misorientation angle distribution data of Fig. 6(c,d). Here, boundaries having misorientation angles of 60° are considered as twin boundaries^25^,^26^. The number fraction of twin boundary is twice higher in the TWIP/LC interfacial region than in the central TWIP region.

The higher fraction of deformation twin in the TWIP/LC interfacial region can be explained by the variation in carbon content and the concentration of deformation in the interfacial area. Since the TWIP-cored sheets are fabricated by the hot-roll-bonding at 1100 °C~900 °C, a considerable number of carbon atoms are diffused from the TWIP region to the LC region. In the TWIP region near the TWIP/LC interface, thus, the carbon content is reduced, and this reduced carbon content results in the decrease in stacking fault energy (SFE), the activation of twin formation, and the higher fraction of deformation twin^27^,^28^. In view of deformation mechanisms, the deformation can be concentrated in the TWIP/LC interfacial region because of anisotropic mechanical properties of the TWIP and LC sheets^29^. The deformation twinning begins to form from pile-ups of coplanar-slip dislocations^30^, and thus the number of dislocation pile-ups increases in the deformation-concentrated TWIP/LC interfacial region. This implies that the twin formation is activated in the TWIP/LC interfacial region as twinning initiation sites are more populated.

**Figure 7:**
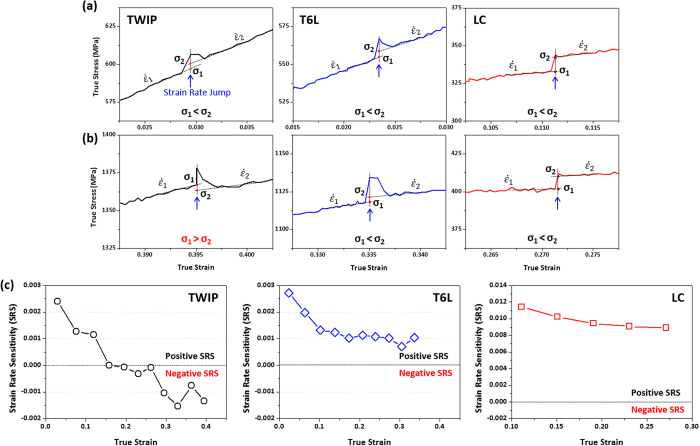
Enlarged stress-strain curves of the strain rate jump test of the TWIP, LC, and T6L sheets in the (**a**) initial and (**b**) later stages of tensile deformation, and (**c**) the variation of SRS as a function of strain. The strain rate sensitivity (SRS) value of the TWIP sheet relatively abruptly decreases with increasing strain, and becomes negative at the strain of 17%. In the LC and T6L sheets, all the SRS values are positive, although they are slowly decreased with increasing strain.

This activation of twinning in the TWIP/LC interfacial region influences the serrated flow behavior. Serrations are closely related with localized bands, as shown in Fig. 5(a,b), which are readily formed by dynamic strain aging when the strain rate sensitivity (SRS) has a negative value^31^,^32^,^33^. The SRS is generally measured from a strain rate jump test on the basis of the following equation^33^,^34^:


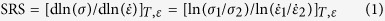


where *σ*, 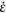, *T*, and *ε* are flow stress, strain rate, temperature, and strain, respectively. The SRSs of the TWIP, LC, and TWIP-cored T6L sheets were measured by varying strain rates from 10^−3^/s to 10^−2^/s during the strain rate jump test, and the results are shown in Fig. 7(a–c). Figure 7(a,b) shows enlarged true stress-strain curves of the strain rate jump test of the TWIP, LC, and T6L sheets in the initial and later stages of tensile deformation, respectively. Their SRS values are positive in the initial stage because the stress increases (σ_1_ < σ_2_) with increasing strain rate (Fig. 7(a)). In the later stage, however, the TWIP sheet has a negative SRS value, whereas positive SRS values are maintained in the LC and T6L sheets (Fig. 7(b)).

It is known that the SRS of material such as LC steel showing dislocation-based plastic deformation is independent of strain or strain rate in a microstructurally stable condition after the sufficient deformation^35^,^36^. However, in TWIP steels, Hadfield steels, Cu alloys, and Al-Mg alloys showing twinning or serrated flow behavior which is known as Portevin Le Chatelier (PLC) effect, the SRS can be dependent on strain or strain rate, and a negative SRS can be shown^37^,^38^,^39^,^40^. Bintu *et al*.^37^ reported that the SRS of an Fe-0.6C-18Mn-0.22Si-1.5Al TWIP steel gradually decreased from 0.002 to −0.002 as the true strain increased to 0.4. In the study on Hadfield steel and Al-Mg alloy^39^,^40^, furthermore, the negative SRS was obtained when they showed the serrated flow in stress-strain curves. Shen *et al*.^38^ reported that a cause of strain dependency on SRS was attributed to the microstructural evolution related with the increased twin fraction and thickness. The mechanism of decreased SRS has not been elucidated yet, but many studies have focused on an interaction between twin boundaries and dislocations according to the increased twin fraction and thickness.

Figure 7(c) shows the variation of SRS as a function of strain. The SRS of the TWIP sheet relatively abruptly decreases from 0.002 to −0.002 with increasing strain, and becomes negative at the strain of 0.15, which is reasonably corresponded with the previous study of Bintu *et al*.^37^,^41^. In the LC and T6L sheets, all the SRS values are positive, and are almost constant except in the initial strain region. The SRS of LC steel is about 0.009, and almost coincides with the previous study of Larour *et al*.^42^. Though deformation twins are well developed in the central TWIP region as well as in the TWIP/LC interfacial region, highly positive SRS values in the LC region lead to the positive SRS in the T6L sheet, and thus serrations do not occur in its stress-strain curve (Fig. 2(a)).

Since the TWIP/LC interface in the TWIP-cored sheets is well bonded without any defects, the yield point is eliminated by the high strain hardening effect in the initial stage of deformation. In the later stage of deformation, the positive SRS values are obtained by the increase in deformation twin density due to low carbon content, low SFE, and strain concentration in the interfacial region. Even when the deformation proceeds further, pearlites existed in the interfacial region are well maintained without forming microvoids or microcracks (Fig. 3(c,d)), and the formation of localized bands is restrained by keeping positive SRS values. These results indicate that the high hardening in the TWIP-cored region and the active twin formation in the TWIP/LC interfacial region beneficially affects the homogeneous deformation in the TWIP-cored sheets without any yield point phenomenon and serrations, respectively. In fact, the mono-layer TWIP and LC steel sheets basically show a drawback of this formation of localized bands, which negatively influences the formability and surface quality. On the other hand, the TWIP-cored sheets show the overall homogenous deformation behavior by complimenting drawbacks of mono-layer TWIP and LC steel sheets, while fully taking advantage of each steel and achieving a wide range of excellent mechanical properties.

### Comparison of measured and calculated tensile properties by a rule of mixtures

**Figure 8:**
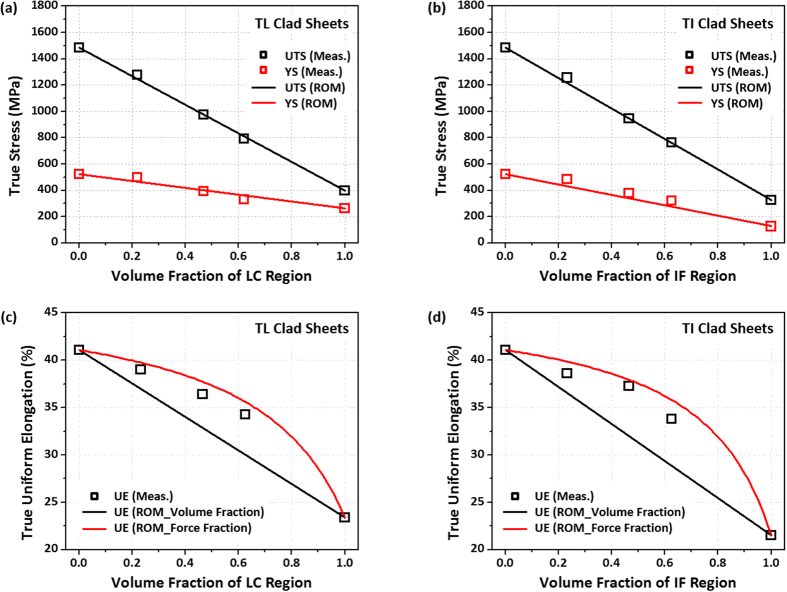
Comparison of true yield strength, ultimate tensile strength, and uniform elongation calculated by a rule of mixtures based on volume fraction or force fraction^34^ with the measured ones for the (**a**,**c**) TL and (**b**,**d**) TI sheets. The measured true strengths and elongation are well matched with the ones calculated by the rule of mixtures based on volume fraction and force fraction, respectively.

Figure 8(a–d) shows the true yield strength, ultimate tensile strength, and uniform elongation calculated by a rule of mixtures based on volume fraction or force fraction for the TWIP-cored TL and TI sheets. The calculated yield and tensile strengths of the TL sheets are well matched with the measured ones (Fig. 8(a)). In the TI sheets, the calculated strengths are also corresponded with the measured ones, although the former tends to be slightly lower than the latter (Fig. 8(b)). However, the measured true uniform elongation does not match with the rule of mixtures based on volume fraction, unlike the cases of yield and tensile strengths, as indicated by black solid lines in Fig. 8(c,d). Thus, the true uniform elongation (ε(u)) is calculated by a rule of mixtures based on force fraction^43^, *i*.*e*.,


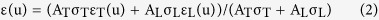


Here, subscripts T and L refer to as TWIP and LC steels, respectively, and A_T_ and A_L_ are cross-sectional-area fractions of the TWIP and LC steels, respectively. The calculated true uniform elongations of the TWIP-cored sheets, as indicated by red solid lines in Fig. 8(c,d), are well matched with the measured ones. This is because the rule of mixtures based on force fraction considers the strain instability of each material of the TWIP-cored sheets^44^. This good correspondence between measured and calculated tensile properties also confirms the excellent interfacial bonding.

The present study on hot-roll-bonding of TWIP steel sheets with thin low-cost LC or IF steel sheets could prove a good way to successfully fabricate TWIP-cored three-layer steel sheets having excellent tensile properties. The TWIP-cored sheets well satisfy a rule of mixtures as the TWIP-cored region dominates overall properties of the TWIP-cored sheets without yield point and serrations in their stress-strain curves. These results are outstanding ones, which have not been reported in previous studies^17^,^31^,^44^,^45^, and the improvement of tensile properties is explained by the strongly bonded interfaces and active twin formation in the interfacial region. Since the fabrication method is actually involved in commercial fabrication processes of hot rolling, any additional processing facilities are not needed. In addition, they can cover a wide range of yield strength, tensile strength, and ductility levels, *e*.*g*., 320~498 MPa, 545~878 MPa, and 48~54%, respectively, requiring in automotive steel sheets by controlling the volume fraction of TWIP-cored region. These TWIP-cored sheets have outstanding tensile properties and economic advantages as well, and thus present new applications to multi-functional automotive steel sheets requiring excellent formability, weldability, Zn coating, delayed fracture properties, and surface quality as well as mechanical properties. In order to further enhance microstructures and properties of TWIP-cored sheets, intensive studies on new designs of alloys or cored sheets for further enhancing formability, weldability, and productivity, and on mechanisms involved in improved properties should be continued in the future.

## Conclusions

TWIP-cored three-layer steel sheets containing thin low-carbon (LC) and interstitial-free (IF) steel surface layers were fabricated by hot-roll-bonding, and their tensile properties and flow behavior were investigated in relation with interfacial microstructural evolutions.

1. When upper and lower surfaces of the TWIP steel substrate were bonded by the hot rolling with LC or IF steel sheets, TWIP/LC or TWIP/IF interfaces were strongly bonded without pores or voids. After the tensile deformation of these TWIP-cored sheets, a few microcracks were formed in thin interfacial pearlites beneath the fracture surface, but were not found in the areas distant from the fracture surface. Because of strong interfacial bonding, the strengths and elongation of the TWIP-cored sheets increased as the volume (or thickness) fraction of TWIP-cored region increased.
2. All the TWIP-cored sheets were homogeneously deformed without any yield point or serrations in their stress-strain curves. According to digital images of strain obtained from a vision strain gauge system for the LC sheet, localized bands appeared in the initial deformation stage, and were joined together to reach the almost same strain throughout the gage section. In the TWIP-cored sheets, however, localized bands were not formed because of very high strain hardening effect of the TWIP steel region in the initial deformation stage as well as strong TWIP/LC interfacial bonding.
3. The digital image correlation analyses of the TWIP sheet indicated that a localized band appeared at the lower region of the gage section, and moved upward as the strain increased. The strains of appearance of localized bands were well matched with serration points in the stress-strain curve, which indicated that serrations were closely related with the localized band formation. In the TWIP-cored sheets, positive strain rate sensitivity values were obtained by the increase in deformation twin density due to low carbon content, low stacking fault energy, and strain concentration in the interfacial region as well as the effect of positive SRS values in the LC steel region, thereby leading to the elimination of localized bands or serrations.
4. Both mono-layer TWIP and LC steel sheets basically showed a drawback of formation of localized bands, which deteriorated the formability and surface quality. On the other hand, the TWIP-cored sheets showed the overall homogenous deformation behavior by complimenting drawbacks of mono-layer TWIP and LC steel sheets, while fully taking advantage of each steel and achieving a wide range of excellent mechanical properties. The active twin formation in the TWIP/LC interfacial region also beneficially affected the homogeneous deformation without any serrations occurring in the TWIP sheet and yield point phenomenon occurring in the LC sheet.
5. In the TWIP-cored sheets, the true yield and tensile strengths calculated by a rule of mixtures based on volume fraction were well matched with the measured strengths. However, the measured true uniform elongation did not match with the rule of mixtures based on volume fraction, but matched with the rule of mixtures based on force fraction because it considered the strain instability of each material of the TWIP-cored sheets. This good correspondence between measured and calculated tensile properties confirmed the excellent interfacial bonding.

## Method

### Fabrication of TWIP-cored three-layer steel sheets

Chemical compositions of commercial TWIP, LC, and IF steels are Fe-0.6C-15Mn-1.2Al, Fe-0.03C-0.2Mn-0.04Al, and Fe-0.002C-0.1Mn-0.04Al-0.03Ti-0.01 Nb (wt.%), respectively. After the TWIP steel plate was surrounded by two LC or IF steel plates, edges of the plates were welded to prepare steel-cored stacks. Stacking ratios of LC (or IF):TWIP:LC(or IF) were controlled to be 1:1:1, 1:2:1, and 1:6:1. These steel stacks were homogenized at 1200 °C for 1 hour, and were hot-rolled at 1100 °C~900 °C with a reduction ratio of 94% to fabricate 2.5-mm-thick TWIP-cored three-layer steel sheets, as schematically illustrated. They were then cooled in a furnace from 450 °C after holding at this temperature for 1 hour in order to simulate a coiling procedure. The three-layer LC/TWIP/LC and IF/TWIP/IF sheets are referred to as ‘TL’ and ‘TI’ sheets, respectively, and numbers 1, 2, and 6 indicate the stacking ratio of TWIP *vs* LC or IF. For example, the ‘T2L’ and ‘T2I’ sheets mean the LC/TWIP/LC and IF/TWIP/IF sheets, respectively, having stacking ratio of 1:2:1. The original stacking ratios were slightly changed after the hot rolling because the TWIP steel was stronger than the LC or IF steel. The 2.5-mm-thick TWIP, LC, and IF steel sheets were also fabricated by the same fabrication route in order to compare with the TWIP-cored sheets.

### Microstructural characterization

The TWIP-cored sheets were mechanically polished and then electro-polished in a solution of CH_3_COOH (92%) and HClO_4_ (8%) by an electro-polisher (model; Lectropol-5, Struers, Denmark) at an operating voltage of 32 V. The polished specimens were etched in a 2% nital solution or a 10% K_2_S_2_O_5_ + 90% distilled water, and microstructures of longitudinal-short-transverse (L-S) plane were observed by an optical microscope (model: Leica DM4000, Wetzlar, Germany) and a field emission scanning electron microscope (FE-SEM, model; XL30S FEG, Philips FEI, USA). Electron back-scatter diffraction (EBSD) analysis (step size; 100 nm) was conducted on the TWIP-cored sheets by the FE-SEM. The data were then interpreted by an orientation imaging microscopy analysis software provided by TexSEM Laboratories (Provo, UT, USA), Inc. Transmission electron microscopy (TEM) specimens were prepared by a focused ion beam (FIB; Helios, FEI, USA) technique, and were observed by a TEM (JEM-2100F, JEOL, Japan) operating at an accelerating voltage of 200 kV.

### Tensile test and digital image correlation

Plate-type tensile specimens (gage length; 25 mm, gage width; 6 mm, gage thickness; 2.5 mm) were prepared along the longitudinal direction. They were tested at room temperature at a strain rate of 10^−3^ s^−1^ by a universal testing machine (model; Instron 5582, Instron Corp., Canton, MA, USA) of 98 kN capacity. During the tensile tests, strain was measured over a gauge length of 25 mm by using a contact 25-mm extensometer. The 0.2% offset stress was determined to be the yield strength in the specimens showing continuous yielding behavior, whereas the lower yield point was determined to be the yield strength in the specimens showing discontinuous yielding behavior. The tensile test was conducted three times for each datum point. Strain or strain rate distributions were measured by a vision strain gauge system (model; ARAMIS v6.1, GOM Optical Measuring Techniques, Germany), which could detect 3-dimensional coordinates of a deforming specimen surface on the basis of digital image processing delivering 3-dimensional displacement and strain^46^. This ARAMIS system recognized the surface structure in digital camera images, and allocated coordinates to image pixels. The local displacement, strain, and strain rate according to the position from the base line (the lower part of the gage section) were measured along the center line of the gage section. Then, the strain or strain rate of deformation bands formed in the gage section was investigated during the tensile deformation.

## Additional Information

**How to cite this article**: Park, J. *et al*. Tensile property improvement of TWIP-cored three-layer steel sheets fabricated by hot-roll-bonding with low-carbon steel or interstitial-free steel. *Sci. Rep.* **7**, 40231; doi: 10.1038/srep40231 (2017).

**Publisher's note:** Springer Nature remains neutral with regard to jurisdictional claims in published maps and institutional affiliations.

## Figures and Tables

**Table 1:** Grain size and room-temperature tensile test results of the TWIP, LC, and IF steel sheets, and the TWIP-cored TL and TI sheets.

Steel Sheet	Austenite Grain Size (μm)	Ferrite Grain Size (μm)	Yield Strength (MPa)	Tensile Strength (MPa)	Uniform Elongation (%)	Total Elongation (%)
TWIP	11.7 ± 3.8	—	523 ± 2	985 ± 1	50.8 ± 2.5	56.4 ± 2.5
LC	—	9.1 ± 3.5	265 ± 3	316 ± 3	26.4 ± 2.6	45.5 ± 2.8
IF	—	34.0 ± 11.9	126 ± 2	264 ± 2	24.0 ± 2.7	45.1 ± 2.6
T1L	11.3 ± 3.7	10.4 ± 4.2	330 ± 11	562 ± 16	40.9 ± 0.3	48.3 ± 0.3
T2L	11.3 ± 3.6	10.4 ± 3.9	393 ± 8	678 ± 22	43.9 ± 0.2	51.8 ± 0.1
T6L	11.6 ± 3.9	10.8 ± 4.2	498 ± 4	878 ± 13	47.7 ± 1.5	54.3 ± 1.5
T1I	10.7 ± 3.7	26.8 ± 6.9	320 ± 4	545 ± 10	40.2 ± 2.9	48.9 ± 1.0
T2I	11.1 ± 3.9	26.8 ± 5.7	379 ± 2	653 ± 23	45.2 ± 2.9	51.1 ± 1.0
T6I	12.1 ± 4.2	27.0 ± 6.1	486 ± 1	854 ± 16	47.1 ± 1.9	53.6 ± 1.4
